# Occupational exposure to endocrine disrupting substances and the risk of breast Cancer: the Singapore Chinese health study

**DOI:** 10.1186/s12889-018-5862-2

**Published:** 2018-07-28

**Authors:** Teofilia Acheampong, Jian-Min Yuan, Woon Puay Koh, Aizhen Jin, Andrew Odegaard

**Affiliations:** 1 Department of Epidemiology, University of California Irvine, School of Medicine, 224 Irvine Hall, Irvine, CA 92697 United States; 2 University of Pittsburgh, University of Pittsburgh Medical Center (Shadyside) Cancer Pavilion, 5150 Centre Avenue, Pittsburgh, PA 15232 United States; 3 Health Services and Systems Research, Duke-NUS Medical School, 8 College Road, Singapore, 169857 Singapore; 4 Saw Swee Hock School of Public Health, National University of Singapore, 12 Science Drive 2, Singapore, 117549 Singapore

**Keywords:** Endocrine disruptors, Occupational exposure, Breast cancer risk, Prospective population-based cohort

## Abstract

**Background:**

Evidence from basic research links exposure to endocrine disrupting chemicals (EDCs) with a higher risk for breast cancer. However, there is less evidence from observational epidemiological research and the results are equivocal. Therefore, we examined the association between occupational exposure to substances where exposure to EDCs is likely and the risk of breast cancer.

**Methods:**

A prospective study consisting of a population-based cohort of 33,458 Singaporean Chinese women aged 45–74 years enrolled in the Singapore Chinese Health Study (SCHS) from 1993 to 98 and followed through 2014. Subjects’ self-reported occupational exposure and duration to industries, job titles, and substance types were garnered at baseline, and cases of incident breast cancer (*N* = 988) were determined by linkage with the Singapore Cancer Registry. Hazard ratios (HR) and 95% confidence intervals (CI) were estimated for exposure to substances, job titles, and industries.

**Results:**

There was no association between cumulative exposure to substances via occupation where EDC exposure is likely and risk of breast cancer. These results were consistent for hypothesized high (HR 0.94, 95% CI: 0.66–1.35), medium (HR 1.03 95% CI: 0.77–1.38) and low (HR 0.74, 95% CI 0.48–1.13) combined substance exposure groups when compared with those who were not exposed via occupation. Similar null associations were observed when examining job titles and industry categories.

**Conclusions:**

There was no association between EDC related occupational exposures and breast cancer risk in working women of the Singaporean Chinese Health Study. Future studies that employ rigorous methods with regard to exposure assessment of EDCs are needed.

**Electronic supplementary material:**

The online version of this article (10.1186/s12889-018-5862-2) contains supplementary material, which is available to authorized users.

## Background

Endocrine disrupting chemicals (EDCs) refer to exogenous substances that can interact with an organism’s endocrine system or general hormonal action [1–3]. These chemicals are often found in: pesticides, fungicides, disinfectants, adhesives, lubricants, chemical solvents, cleaning agents, dyes, paints, oils, tubing, wiring, plastics, plasticizers, coal or coal combustion and heavy metals [1, 4–8]. Exposure to EDCs has become pervasive globally, and there is evidence that they influence biological pathways which may increase cancer risk [3, 9–11]. Moreover, there is a particular concern for people who work within an occupation or industry where even greater EDC exposure may occur, potentially explaining differential rates of cancer within certain occupations [12–14].

It has been hypothesized that part of the increase in the incidence of hormone related cancers, such as breast cancer, could be the result of the increased presence of EDCs in the environment due to westernization and industrialization [15–17]. Yet previous epidemiological studies examining the association between exposure to substances that may have hormonal activity and risk for breast cancer have been inconsistent [18–25]. Specifically, positive associations were found for different exposures in some US and Canadian populations when analyses assessed interactions by reproductive risk factors [19, 20, 23]. However other large US and European studies have found no associations [18, 21, 22, 24, 25]. This highlights the scientific utility of examining populations with varying underlying breast cancer risk and reproductive patterns than those of western and European countries. Furthermore, in terms of race ethnicity, the previous studies are largely non-Hispanic white. Therefore, research utilizing an Eastern population of Chinese women in Singapore, a country historically known for its industrial history [26], is distinct and could make a novel contribution to the topic.

Our primary aim was to examine the association of EDC related occupational exposures with the risk of breast cancer in women. We hypothesized that a proxy for higher composite exposure to EDC via occupational exposures was associated with higher breast cancer risk.

## Methods

### Study population

The design of the SCHS has been previously described [27]. Briefly, the cohort was drawn from men and women, from 45 to 74 years of age (mean age, 56.5 years), who belonged to one of the major dialect groups (Hokkien or Cantonese) of Chinese in Singapore. Between April 1993 and December 1998, 63,257 individuals completed an in-person interview. The questionnaire obtained information on diet, demographics, physical activity, reproductive history (on women only) and medical history. In addition, there was an assessment of occupational history and exposures. Two follow-ups were conducted over the last 23 years. This analysis was restricted to women without a diagnosis of invasive breast cancer at baseline (*N* = 34,028), with complete data (*N* = 33,458), after excluding participants with incomplete data regarding diet pattern (*n* = 570). Written, informed consent was obtained from all study participants. Institutional review boards at the National University of Singapore, University of Pittsburgh, and the University of California, Irvine approved this study.

### Assessment of occupational exposure and covariates

At the baseline interview participants self-reported lifetime exposure through their occupations. The job titles included, “Welder, Textile Machine Mechanic, Other Mechanic, Cotton Spinner or Weaver, Painter, Textile Dyer, Machinist, Printer, Tailor or Seamstress, Janitor or Cleaner, Vocational Driver, Food Hawker, Cook or Kitchen Worker” [28]. The industries included, “manufacture and repair of boots, shoes or other leather goods, manufacture of furniture and cabinets, cotton textile, electrical and electronic industry, lumber and sawmill, carpentry or joinery, rubber and tire manufacturing, manufacture of dyes or dyestuffs, manufacture of paints, manufacture of plastics, petroleum, metal production or processing, construction work, urea formaldehyde manufacturing” [28]. Lastly, the substances listed were “cotton dust, wood dust, wood preservatives, metal dust or fumes, rock or mineral dust, smoke (all types), smoke from welding, smoke from burning coal or coke, smoke from burning wood, other smoke, pesticides, asbestos, coal tar, soot, pitch, acid or alkali solutions, chemical solvents, dyes or dyestuffs, cutting, cooling or lubricating oils, paints, or formaldehyde” [28]. For all of the aforementioned exposures lasting one year or longer, participants reported the duration of exposure as: 1–4 years, 5–9 years, 10–14 years, 15–19 years, and ≥ 20 years [28].

A priori group classification of EDC related occupational exposures was identified from the literature and this included: Chlorophenol, heavy metals, polycyclic aromatic hydrocarbons (PAH), pesticides, organic solvents, dyes, bisphenol A, phthalates, synthetic resins, alkylphenols. PCBs, phenylphenol, and brominated flame retardants. To test the study hypothesis, substances from the above list were assigned to the EDC group list as probable exposure to EDCs based upon literature evidence; the analysis was repeated for the industry categories, as well as the job title categories. The details of these groupings as well as the references for the selections are presented in Additional file 1: Table S1.

Covariate information was ascertained from the baseline interview. This included age, dialect, educational attainment, smoking habits, alcohol use, body mass index (kg/m^2^), contraception history, hormone therapy use, reproductive factors, physical activity and a vegetable-fruit-soy rich dietary pattern score [29].

### Breast Cancer ascertainment

Incident primary invasive breast cancer cases as well as deaths were ascertained through record linkage with the population-based Singapore Cancer Registry and the Singapore Registry of Births and Deaths through 2014. A unique national identification number given to all Singaporeans was used for linkage. Thus far, only *n* = 52 participants (0.08%) were lost to follow-up due to migration out of Singapore. This suggests that emigration within this study population is negligible and vital statistics at follow-up was essentially complete.

### Statistical methods

To test whether greater exposure to occupational related EDCs is associated with higher risk for breast cancer we created an index from the occupational exposure assessment as described in Additional file 1: Table S1. For each participant the index for the substance was a function of the number and duration of exposures. For any substance participants reported exposure to as one year or longer the median of those categories was assigned as duration. Specifically, for 1–4 years the median was 2.5, for 5–9 years the median assigned was 7, for 10–14 years the median assigned was 12, for 15–19 years the median was 17 and for ≥20 years we assigned 22. The index is thus the sum of the quantity x median duration (years) of each individual exposure for the reported substance and reflects the estimated number of years of exposure to occupational related EDCs. This approach was repeated for reported job titles, and industry categories.

Participants with a non-zero exposure index score were ranked into tertiles. Women who reported no occupational exposure to any of the substances, jobs or industries were another category (*N* = 18,138). Lastly, women who reported working in an industry, job title, or reported occupational exposure to a specific substance that did not fit the a priori definition for likely EDC exposure were defined as a category as well.

Cox regression models were used to estimate the Hazard Ratios and corresponding 95% confidence intervals for the association between the occupational exposure groups and incident breast cancer. The referent group was women who reported no occupational exposure to any industry, job, or substance (*N* = 18,138). For each study subject, person-years were counted from the date of the baseline interview to the date of incident breast cancer diagnosis, death, date of last contact (for the few subjects who migrated out of Singapore), or 31 December 2014, whichever occurred first. There was no evidence that proportional hazards assumptions were violated as indicated by the lack of a interaction between indices and a function of the survival time in models, and by viewing log (−log (survival)) plots.

Two models were constructed to examine the association between likely occupational exposure to EDCs and risk of breast cancer during follow up. Model 1 included age at baseline, dialect (Cantonese or Hokkiens), year enrolled (between 1993 and 1995 vs 1996–1998), educational attainment (no formal education/primary/secondary/beyond secondary), smoking habits (never/former/current), alcohol use (never/ever), body mass index (kg/m^2^), contraception history (never/former/current), hormone therapy use (never/former/current), physical activity (time in strenuous sports and/or vigorous work) and a vegetable-fruit-soy rich dietary pattern score; [29] model 2 further included reproductive factors such as parity vs nulliparous, age at first birth (< =20, 21–25, 26–30, 31–35, > 35), menarche age (< 11 years, 11–14, 15–16, ≥17) and menopausal status. We tested for effect modification by recognized risk factors for breast cancer, specifically BMI categories (< 23.0 kg/m^2^ versus ≥23.0 kg/m^2^), smoking (ever/former/current), alcohol use (never/ever), menopausal status (pre-menopause/menopause), and age group (45–55, 55–65, 65–74). We also carried out a sensitivity analysis to inform the interpretation of our a priori groupings by examining specific substance types, job titles, and industry of potential occupational EDC exposure. All analyses were performed with SAS version 9.3 (SAS Institute, Cary, NC).

## Results

As of December 31, 2014, 2.95% of the study population developed a primary invasive breast cancer (*N* = 988). Descriptive participant characteristics at baseline for the different exposure categories are presented in Table 1 and Additional file 1: Tables S2 and S3, across exposure indices. In contrast to participants with no exposure via job substance or occupation, those with greater exposure were slightly younger, had less education, a history of smoking and drinking, were more so of Cantonese ancestry, and nulliparous.

**Table 1:** Baseline Characteristics of Participants According to Combined Potential EDC Substance Groups: The Singapore Chinese Health Study

	No Exposure to Substance, Industry & Job (*N* = 18,138)		No Substance Reported (*N* = 11,524)		Tertile 1 (*N* = 1023)		Tertile 2 (*N* = 1631)		Tertile 3 (*N* = 1142)	
	Mean	± SD	Mean	± SD	Mean	± SD	Mean	± SD	Mean	± SD
Covariates										
Age at baseline	57	8.2	55	7.7	54	7.3	55	7.4	56	7.9
BMI	23	3.2	23	3.4	23	3.5	23	3.7	23	3.47
	N	(%)	N	(%)	N	(%)	N	(%)	N	(%)
Education										
No formal Education	7379	40.7	4577	39.7	318	31.1	640	39.2	495	43.4
Primary/Secondary	9929	54.7	6872	59.6	694	67.8	974	59.7	628	55.0
Beyond Secondary	830	4.6	75	0.7	11	1.1	17	1.0	19	1.7
Dialect										
Hokkiens	10,378	57.2	5335	46.3	477	46.6	711	43.6	505	44.2
Alcohol Consumption										
Never drinker	16,777	92.5	10,338	89.7	906	88.6	1426	87.4	998	87.4
Smoking status										
Never	16,677	92.0	10,510	91.2	926	90.5	1452	89.0	999	87.5
Menopause Status										
Menopausal	13,457	74.2	7988	69.3	643	62.9	1113	68.2	812	71.1
Age at Menarche										
< =14 years of age	9575	52.8	6073	52.7	589	57.6	851	52.2	562	49.2
15–16 years of age	6283	34.6	3982	34.6	328	32.1	548	33.6	401	35.1
> = 17 years of age	2280	12.6	1469	12.8	106	10.4	232	14.2	179	15.7
Parity										
At least 1 child birth	16,917	93.3	10,662	92.5	964	94.2	1532	93.9	1016	89.0
Age at First Birth										
< =20 years of Age	5010	27.6	2848	24.7	238	23.3	392	24.0	337	29.5
21–30 years of age	11,495	63.4	7390	64.1	686	67.1	1063	65.2	671	58.8
> = 31 years of age	1633	9.0	1286	11.2	99	9.7	176	10.8	134	11.7
Birth Control Use										
Never	13,814	76.2	8194	71.1	624	61.0	1134	69.5	832	72.9
Ever Hormone Use										
Never Estrogen	17,151	94.6	10,941	94.9	949	92.8	1520	93.2	1052	92.1
Never Progesterone	17,865	98.5	11,354	98.5	1004	98.1	1608	98.6	1111	97.3

The results from the Cox regression analysis for the 3 different indices of potential occupational exposure to EDCs and risk for breast cancer are presented in Table 2. We observed no association between the occupational industry, job title, or substance index and risk for breast cancer. There was no evidence that these null results differed by any of our a priori tests for effect modification by known breast cancer risk factors (data not shown). The results also did not differ in a sensitivity analysis that utilized participants who reported a history of occupational exposures but unlikely EDC exposure as the referent group (data not shown). Lastly, Additional file 1: Table S4 presents the results for the sensitivity analysis examining individual categories of industry, job title, and substance exposure via occupation. Similar to the main index results, there was no association between any of the individual categories of industry, job title or substance with risk for developing breast cancer.

**Table 2:** Hazard Ratios and 95% CI for breast cancer according to EDC exposure level for each occupational domain: The Singapore Chinese Health Study

		Model 1^a^		Model 2^b^	
Parameter	Cases % (Case N/ Overall N)	HR	95% CI	HR	95%CI
Median (IQR) of Substance Index: 0 (0–2.5)					
No Exposure through job/substance/industry	3.0% (550/18,138)	1	Ref	1	Ref
No Exposure to substance type	2.8% (334/11,524)	0.95	0.83–1.10	0.95	0.82–1.09
Tertile 1 (Substance)	2.2% (22/1023)	0.74	0.48–1.14	0.74	0.48–1.13
Tertile 2 (Substance)	3.0% (50/1631)	1.05	0.78–1.40	1.03	0.77–1.38
Tertile 3 (Substance)	2.8% (32/1142)	0.95	0.66–1.36	0.94	0.66–1.35
Median (IQR) of Job Title Index: 0 (0–0)					
No Exposure through job/substance/industry	3.0% (550/18,138)	1	Ref	1	Ref
No Exposure via Job Title	3.0% (134/4381)	1.02	0.84–1.24	1.02	0.84–1.23
Tertile 1 (Job Title)	2.4% (75/3039)	0.84	0.66–1.08	0.84	0.66–1.07
Tertile 2 (Job Title)	3.0% (124/4165)	0.98	0.80–1.20	0.97	0.80–1.19
Tertile 3 (Job Title)	2.8% (105/3735)	0.92	0.74–1.13	0.91	0.74–1.13
Median (IQR) of Industry Index: 0 (0–0)					
No Exposure through job/substance/industry	3.0% 550/18,138	1	Ref	1	Ref
No Exposure via Industry type	2.7% 263/9635	0.91	0.78–1.05	0.91	.78–1.05
Tertile 1 (Industry)	3.1% 61/1939	1.05	0.80–1.37	1.05	0.80–1.37
Tertile 2 (Industry)	2.7% 42/1579	0.88	0.64–1.21	0.87	0.64–1.20
Tertile 3 (Industry)	3.3% 72/2167	1.08	0.84–1.38	1.07	0.83–1.38

*The index = the sum of the quantity x median duration of years of each individual exposure for the reported substance and reflects the estimated number of years of exposure to occupational related EDCs. Those with non-zero scores were ranked into tertiles

^a^Model 1 included age, dialect, enrollment year, education, smoking, alcohol use, BMI, birth control use, hormone therapy use, physical activity, dietary pattern score

^b^Model 2: Model 1 + parity, age at first birth, menarche age and menopausal status

## Discussion

In this large, prospective study of Chinese Singaporean women there was no association between EDC related occupational exposures via industry, specific jobs, or specific substances, and risk for invasive breast cancer.

This is the first, large, population-based study to examine occupational exposures and breast cancer risk in a Singaporean Chinese cohort. The majority of studies have utilized self-reported exposure to particular chemical classes and job titles, and our null findings are consistent with some previous reports [20, 24, 25]. However, other studies reported sub-group or sensitivity analysis findings of note. A study done by Ekenga et al. [19] examined occupational exposure to solvents and found that risk of invasive breast cancer was not associated with lifetime exposure to solvents. However amongst parous women, models stratified by solvent exposure (yes vs. no), estimated a 39% increased risk of ER+ invasive breast cancer in comparison to women who never worked with solvents (HR, 1.39; 95% CI, 1.03–1.86) [19]. Furthermore, a Canadian population-based case-control study found that overall, self-reported exposure to endocrine disruptors was associated with increased odds of breast cancer for women with 10 years exposure or more in: Agriculture, automotive plastics manufacturing, food canning, and metalworking [23].

The aforementioned studies were able to assess breast cancer molecular subtypes, as well as reproductive risk factors. This study did not observe differences in risk due to exposure by reproductive characteristics. Furthermore, we were not able to carry out sensitivity analyses related to breast cancer molecular subtypes. This is a potential limitation to consider in the interpretation of this study’s results since the mechanisms leading to different molecular subtypes of breast cancer may be differentially impacted by EDC exposure. Lastly, we did not find meaningful differences based on BMI, smoking status, alcohol menopausal status or age group. Two other studies did assess some of these domains as well, and their results were similar to this analysis [20, 22].

This study makes an important contribution to the literature as it presents results from a Singaporean Chinese population, and the broader research base has limited evidence from non-White populations. In general, the age standardized incidence rates of breast cancer in the Asia-Pacific region, are markedly lower in comparison to Europe and the US [30–32]. However, Singapore (particularly the Chinese population) has the highest rates in East and Southeast Asia, and rates have increased by 3–4% per year with Singaporean Chinese women having the highest rates within the country [32, 33]. Prior studies [33–35] have proposed potential reasons behind the increasing breast cancer risk in this population. Some are potentially related to birth cohort differences due to the influence of westernization and industrialization [35]. Historically, there has been a change in reproductive behaviors (e.g. a decrease in parity, breastfeeding, and fertility rates), [33, 34, 36, 37] and an increase in adiposity (e.g. central adiposity) [36, 38]. Lastly, the rapid industrialization of Singapore during the 1960s and 70s increased the prevalence of women entering the labor market for manufacturing & commercial jobs almost 2-fold during that time period [39]. Hence, occupational studies within this particular population are needed and relevant.

Another important aspect to consider related to the incidence of invasive breast cancer in this cohort, is that the largest burden of cases occurs in women aged 40–50, this distribution is well documented in this population [40]. Due to the aforementioned trends in breast cancer incidence in this population, and the greater burden in the younger portion of the SCHS cohort, it is important to consider the potential for other unmeasured or structural factors related to age that might confound the results. However, our attempt to address this through stratified analyses by age group did not present evidence to support differential results by age.

Overall, there are a number of strengths and limitations to consider while interpreting the results of this study. For one, this is a large prospective population-based cohort study, with a novel population, relevant occupational assessment, and essentially complete follow up. Furthermore, the extensive reproductive, medical, socio-demographic, and lifestyle assessments provided the basis to carry out extensive analyses adjusting for a range of possible confounders. Also, utilizing a population-based cohort to conduct an occupational analysis versus an occupational cohort further minimizes selection bias related to the health worker affect [28, 41]. On the other hand, occupational exposures were self-reported, and only serve as a proxy for EDC exposures, which were the hypothesized carcinogens. The sensitivity analyses were designed to inform whether the composite indices created were potentially misclassified, however other exposure assessment (via industries and jobs) analyses were also null. The potential consequences of many exposures are likely to depend on context, timing, type, and most of all dosage of the exposure [42]. However, due to the low prevalence for each exposure, this study had limited ability to examine these details. Also, assessing exposures during any time point of the lifespan could lead to non-differential miss-classification. Thus, these results provide evidence on the general occupation, industry, and substance level for an association with breast cancer risk that is null, but only weak evidence due to misclassification on a-priori groupings intended to inform EDC exposure via occupation and breast cancer risk.

## Conclusions

In summary, the findings of this analysis demonstrate no association between occupation, industry, different substances and risk for breast cancer in working women of the Singaporean Chinese Health Study; furthermore, it does not support the hypothesis that cumulative combined occupational exposure as a proxy for higher exposure to endocrine disruptors is associated with increased breast cancer risk. Future research that incorporates detailed, objective exposure assessment of EDCs across critical points of the lifespan will provide the best opportunity to delineate any causal role of EDCs in breast cancer.

## Additional file

Additional file 1: Supplementary tables and listing of the occupational exposure assessment. (DOCX 98 kb)

## Abbreviations

BMI: Body mass index
CI: Confidence interval
EDCs: Endocrine disrupting chemicals
HR: Hazard ratio
SCHS: Singapore Chinese Health Study
