# Supplementary material for: Occupational exposure to endocrine disrupting substances and the risk of breast Cancer: the Singapore Chinese health study
Source: BMC Public Health. 2018 Jul 28;18:929. doi: 10.1186/s12889-018-5862-2 (PMC6064056; doi:10.1186/s12889-018-5862-2)
Supplement: Supplementary file 1 — Supplementary tables and listing of the occupational exposure assessment. (DOCX 98 kb) [file 12889_2018_5862_MOESM1_ESM.docx]

| **Additional file 1: Table 1. Grouping of occupational exposures for analysis of the SCHS based upon evidence of exposure to potential endocrine-disrupting chemicals (EDC).** | |  |
| --- | --- | --- |
| **OCCUPATIONAL EXPOSURE** | **EDC GROUP ASSIGNMENT** | **REFERENCE** |
| **SUBSTANCES** |  |  |
| WOOD DUST | Chlorophenol, Heavy metals, Polycyclic Aromatic Hydrocarbons (PAH), Pesticides | ^1–6^ |
| WOOD PRESERVATIVES | Heavy metals, PAHs, Pesticides | ^1–4,6^ |
| METAL DUST | Heavy metals, PAH, Organic solvents | ^1,3,8,9^ |
| SMOKE OF ALL TYPES | PAHs | ^1,5^ |
| WELDING SMOKE | Heavy metals, PAH, Organic solvents | ^1,3,8,9^ ^10^ |
| BRNNG COAL SMOKE | PAHs | ^1^ |
| BURNING WOOD SMOKE | PAHs | ^1^ |
| OTHER SMOKE | PAHs | ^1^ |
| PESTICIDES | Pesticides | ^1,11^ |
| COAL/TAR/SOOT | PAHs | ^1,7,12,13,12^ |
| CHEMICAL SOLVENTS | Organic solvents | ^1,14^ |
| DYES/DYESTUFFS | Dyes | ^1,15,16^ |
| CUTTING OILS | PAHs, Heavy metals | ^17,18^ |
| PAINTS | Bisphenol A, Phthalates, Synthetic resins, Organic solvents, Heavy metals | ^14,19,20^ |
| **JOB TITLE** |  |  |
| WELDER | Heavy metals, PAH, Organic solvents | ^2,3,8,9^ |
| TEXTILE MACHINE | Alkylphenols, Synthetic resins, Organic solvents. | ^1,21^ |
| OTHER MECHANIC | Heavy metals, Organic solvents, PAH | ^22,23^ |
| COTTON SPINNER | PAHs, Heavy metals | ^21^ |
| PAINTER | Bisphenol A, Phthalates, Synthetic resins, Organic solvents, Heavy metals | ^1,19,24^ |
| TEXTILE DYER | Phthalates, Heavy metals, Organic solvents, PAH | ^15,16^ |
| MACHINIST | Heavy metals, Organic solvents | ^22^ |
| PRINTER | Phthalates, Bisphenol A, Heavy metals, Organic solvents, PAH | ^14,25^ |
| TAILOR | Organic solvents | ^1^ |
| JANITOR | Organic solvents | ^1,14^ |
| DRIVER | PAHs, Organic solvents | ^26^ ^27^ |
| FOOD HAWKER | PAHs, Heavy metals | ^28^ |
| **INDUSTRY** |  |  |
| MANUFACTURE LEATHER | Chlorophenol, Heavy metals, Organic solvents, Phthalates | ^1,29^ |
| MANUFACTURE FURNITURE | Chlorophenol, Heavy metals, PAHs, Phthalates, Organic solvents | ^1–5,19,20,30^ |
| COTTON TEXTILE | Alkylphenols, Synthetic resins, Organic solvents. Also see Dyes. | ^1,15,16^ |
| MANUFACTURE ELECTRICAL PARTS | PCBs, Organic solvent, Phenylphenol, Bisphenol A, Phthalates, Heavy metals | ^1,31^ |
| LUMBER | Chlorophenol, Heavy metals, PAHs, Pesticides | ^1–5,19,20,30^ |
| CARPENTRY | Chlorophenol, Heavy metals, PAHs, Pesticides | ^1–5,19,20,30^ |
| MANUFACTURE RUBBER | Phenylphenol, Brominated flame retardants, PCB | ^1,32^ |
| MANUFACTURE DYES | Phthalates, Bisphenol A, Heavy metals, Organic solvents, PAH | ^1,14–16,33^ |
| MANUFACTURE PAINT | Bisphenol A, Phthalates, Synthetic resins, Organic solvents, Heavy metals | ^14,19,24,34,35^ |
| MANUFACTURE PLASTCS | Phthalates, Synthetic resins, Bisphenol A, Heavy metals, Organic solvents | ^1^ |
| PETROLEUM | Organic solvents, Heavy metals | ^1,14,23,36^ |
| METAL PRODUCTION | Heavy metals, PAH, Organic solvents, Synthetic resins | ^2,3,8^ ^10^ |
| CONSTRUCTION | Bisphenol A, Phthalates, Synthetic resins, Organic solvents, Heavy metals | ^37^ |

| **Additional file 1: Table 2. Baseline Characteristics of Participants According to Combined Potential EDC Job Title Groups: The Singapore Chinese Health Study** | | | | | | | | | | |
| --- | --- | --- | --- | --- | --- | --- | --- | --- | --- | --- |
|  | **No Exposure to Substance, Industry & Job (N=18,138)** | | **No Job Title reported (N=4,381)** | | **Low Exposure (N=3,039)** | | **Medium Exposure (N=4,165)** | | **High Exposure (N=3,735)** | |
| **Covariates** | **Mean** | **± SD** | **Mean** | **± SD** | **Mean** | **± SD** | **Mean** | **± SD** | **Mean** | **± SD** |
| Age at baseline | 57 | 8.2 | 55 | 8.0 | 55 | 7.5 | 55.0 | 7.3 | 56 | 7.5 |
| BMI | 23 | 3.2 | 23 | 3.4 | 23 | 3.4 | 23.0 | 3.4 | 23 | 3.4 |
|  | **N** | **(%)** | **N** | **(%)** | **N** | **(%)** | **N** | **(%)** | **N** | **(%)** |
| **Education** |  |  |  |  |  |  |  |  |  |  |
| None | 7,379 | 40.7 | 1,730 | 39.5 | 1,200 | 39.5 | 1,606 | 38.6 | 1,494 | 40.0 |
| Primary/Secondary | 9,929 | 54.7 | 2,591 | 59.1 | 1,821 | 59.9 | 2,535 | 60.9 | 2,221 | 59.5 |
| Beyond Secondary | 830 | 4.6 | 60 | 1.4 | 18 | 0.6 | 24 | 0.6 | 20 | 0.5 |
| **Dialect** |  |  |  |  |  |  |  |  |  |  |
| Hokkiens | 10,378 | 57.2 | 2,075 | 47.4 | 1,496 | 49.2 | 1,879 | 45.1 | 1,578 | 42.3 |
| **Alcohol Intake** |  |  |  |  |  |  |  |  |  |  |
| Never drinker | 16,777 | 92.5 | 3,939 | 89.9 | 2,692 | 88.6 | 3,715 | 89.2 | 3,322 | 88.9 |
| **Smoking status** |  |  |  |  |  |  |  |  |  |  |
| Never | 16,677 | 92.0 | 4,002 | 91.4 | 2,732 | 89.9 | 3,786 | 90.9 | 3,367 | 90.2 |
| **Menopause Status** |  |  |  |  |  |  |  |  |  |  |
| Menopausal | 13,457 | 74.2 | 2,736 | 62.5 | 2,106 | 69.3 | 2,890 | 69.4 | 2,824 | 75.6 |
| **Age at Menarche** |  |  |  |  |  |  |  |  |  |  |
| < =14 years of age | 9,575 | 52.8 | 2,301 | 52.5 | 1,627 | 53.5 | 2,205 | 52.9 | 1,942 | 52.0 |
| 15-16 years of age | 6,283 | 34.6 | 1,477 | 33.7 | 1,025 | 33.7 | 1,433 | 34.4 | 1,324 | 35.5 |
| > = 17 years of age | 2,280 | 12.6 | 603 | 13.8 | 387 | 12.7 | 527 | 12.7 | 469 | 12.6 |
| **Parity** |  |  |  |  |  |  |  |  |  |  |
| Parous | 16,917 | 93.3 | 4,061 | 92.7 | 2,895 | 95.3 | 3,886 | 93.3 | 3,332 | 89.2 |
| **Age at First Birth** |  |  |  |  |  |  |  |  |  |  |
| < =20 years of Age | 5,010 | 27.6 | 1,050 | 24.0 | 742 | 24.4 | 1,006 | 24.2 | 1,017 | 27.2 |
| 21-30 years of age | 11,495 | 63.4 | 2,863 | 65.4 | 1,995 | 65.6 | 2,694 | 64.7 | 2,258 | 60.5 |
| >= 31 years of age | 1,633 | 9.0 | 468 | 10.7 | 302 | 9.9 | 465 | 11.2 | 460 | 12.3 |
| **Birth Control Use** |  |  |  |  |  |  |  |  |  |  |
| Never | 13,814 | 76.2 | 3,074 | 70.2 | 2,012 | 66.2 | 2,920 | 70.1 | 2,778 | 74.4 |
| **Hormone Use** |  |  |  |  |  |  |  |  |  |  |
| Never Estrogen | 17,151 | 94.6 | 4,136 | 94.4 | 2,875 | 94.6 | 3,944 | 94.7 | 3,507 | 93.9 |
| Never Progesterone | 17,865 | 98.5 | 4,314 | 98.5 | 2,994 | 98.5 | 4,102 | 98.5 | 3,667 | 98.2 |

| **Additional file 1: Table 3. Baseline Characteristics of Participants According to Combined Potential EDC Industry Groups: The Singapore Chinese Health Study** | | | | | | | | | | |
| --- | --- | --- | --- | --- | --- | --- | --- | --- | --- | --- |
|  | **No Exposure to Substance, Industry & Job (N=18,138)** | | **No Industry Reported (N=9,635)** | | **Low Exposure (N=1,939)** | | **Medium Exposure (N=1,579)** | | **High Exposure (N=2,167)** | |
| **Covariates** | **Mean** | **± SD** | **Mean** | **± SD** | **Mean** | **± SD** | **Mean** | **± SD** | **Mean** | **± SD** |
| Age at baseline | 57 | 8.2 | 56 | 7.5 | 54 | 7.8 | 53 | 7.4 | 55 | 8 |
| BMI | 23 | 3.2 | 23 | 3.4 | 23 | 3.4 | 23 | 3.5 | 23 | 3.4 |
|  | **N** | **(%)** | **N** | **(%)** | **N** | **(%)** | **N** | **(%)** | **N** | **(%)** |
| **Education** |  |  |  |  |  |  |  |  |  |  |
| None | 7,379 | 40.7 | 3,777 | 39.2 | 706 | 36.4 | 607 | 38.4 | 940 | 43.4 |
| Primary/Secondary | 9,929 | 54.7 | 5,784 | 60 | 1,214 | 62.6 | 960 | 60.8 | 1,210 | 55.8 |
| Beyond Secondary | 830 | 4.6 | 74 | 0.8 | 19 | 1 | 12 | 0.8 | 17 | 0.8 |
| **Dialect** |  |  |  |  |  |  |  |  |  |  |
| Hokkiens | 10,378 | 57.2 | 4,407 | 45.7 | 999 | 51.5 | 793 | 50.2 | 829 | 38.3 |
| **Alcohol** |  |  |  |  |  |  |  |  |  |  |
| Never drinker | 16,777 | 92.5 | 8,607 | 89.3 | 1,726 | 89 | 1,413 | 89.5 | 1,922 | 88.7 |
| **Smoking status** |  |  |  |  |  |  |  |  |  |  |
| Never | 16,677 | 92 | 8,718 | 90.5 | 1,780 | 91.8 | 1,453 | 92 | 1,936 | 89.3 |
| **Menopause Status** |  |  |  |  |  |  |  |  |  |  |
| Menopausal | 13,457 | 74.2 | 7,016 | 72.8 | 1,164 | 60 | 914 | 57.9 | 1,462 | 67.5 |
| **Age at Menarche** |  |  |  |  |  |  |  |  |  |  |
| < =14 years of age | 9,575 | 52.8 | 5,086 | 52.8 | 1,062 | 54.8 | 847 | 53.6 | 1,080 | 49.8 |
| 15-16 years of age | 6,283 | 34.6 | 3,329 | 34.6 | 617 | 31.8 | 545 | 34.5 | 768 | 35.4 |
| > = 17 years of age | 2,280 | 12.6 | 1,220 | 12.7 | 260 | 13.4 | 187 | 11.8 | 319 | 14.7 |
| **Parity** |  |  |  |  |  |  |  |  |  |  |
| Parous | 16,917 | 93.3 | 8,916 | 92.5 | 1,823 | 94 | 1,485 | 94.1 | 1,950 | 90 |
| **Age at First Birth** |  |  |  |  |  |  |  |  |  |  |
| < =20 years of Age | 5,010 | 27.6 | 2,462 | 25.6 | 454 | 23.4 | 341 | 21.6 | 558 | 25.8 |
| 21-30 years of age | 11,495 | 63.4 | 6,112 | 63.4 | 1,294 | 66.7 | 1,066 | 67.5 | 1,338 | 61.7 |
| >= 31 years of age | 1,633 | 9 | 1,061 | 11 | 191 | 9.9 | 172 | 10.9 | 271 | 12.5 |
| **Birth Control Use** |  |  |  |  |  |  |  |  |  |  |
| Never | 13,814 | 76.2 | 6,829 | 70.9 | 1,290 | 66.5 | 1,062 | 67.3 | 1,603 | 74 |
| **Hormone Use** |  |  |  |  |  |  |  |  |  |  |
| Never Estrogen | 17,151 | 94.6 | 9,063 | 94.1 | 1,828 | 94.3 | 1,505 | 95.3 | 2,066 | 95.3 |
| Never Progesterone | 17,865 | 98.5 | 9,473 | 98.3 | 1,904 | 98.2 | 1,560 | 98.8 | 2,140 | 98.8 |

| **Additional file 1: Table 4. Hazard Ratio and 95% CI for Breast Cancer According to Individual Potential EDC Exposure: The Singapore Chinese Health Study** | | | | | |
| --- | --- | --- | --- | --- | --- |
|  |  | **Model 1** | | **Model 2** |  |
|  | **Cases (N)/Total** | **HR** | **95%CI** | **HR** | **95%CI** |
| **SUBSTANCE** |  |  |  |  |  |
| **Wood Dust** |  |  |  |  |  |
| No Exposure via any  job/substance/industry | 550 / 18,138 | 1 Ref |  | 1 Ref |  |
| No Exposure to wood dust | 420/14,881 | 0.94 | 0.82 -1.07 | 0.93 | 0.81-1.06 |
| <10 years of Exposure | 11/268 | 1.45 | 0.80-2.64 | 1.44 | 0.79-2.63 |
| >10 years of Exposure | 7/171 | 1.41 | 0.67-2.98 | 1.4 | 0.66-2.96 |
| **Wood Preservatives** |  |  |  |  |  |
| No Exposure via any  job/substance/industry | 550 / 18,138 | 1 Ref |  | 1 Ref |  |
| No Exposure to wood preservative | 437/15,270 | 0.95 | 0.83-1.09 | 0.94 | 0.83-1.08 |
| <10 years of Exposure | 0/28 | 0 | 0.00-0.00 | 0 | 0.00-0.00 |
| >10 years of Exposure | 1/22 | 1.44 | 0.20-10.2 | 1.47 | 0.21-10.5 |
| **Metal Dust** |  |  |  |  |  |
| No Exposure via any  job/substance/industry | 550 / 18,138 | 1 Ref |  | 1 Ref |  |
| No Exposure to metal dust | 418/14,747 | 0.94 | 0.82-1.08 | 0.94 | 0.82-1.07 |
| <10 years of Exposure | 12/351 | 1.2 | 0.67- 2.13 | 1.18 | 0.66-2.09 |
| >10 years of Exposure | 8/222 | 1.2 | 0.60-2.42 | 1.21 | 0.60-2.44 |
| **Smoke from Welding** |  |  |  |  |  |
| No Exposure via any  job/substance/industry | 550 / 18,138 | 1 Ref |  | 1 Ref |  |
| No Exposure to smoke from welding | 433/15,120 | 0.95 | 0.83-1.09 | 0.94 | 0.83-1.08 |
| <10 years of Exposure | 1/123 | 0.28 | 0.04-1.97 | 0.27 | 0.04-1.93 |
| >10 years of Exposure | 4/77 | 1.76 | 0.66-4.73 | 1.73 | 0.64-4.64 |
| **Smoke of all types** |  |  |  |  |  |
| No Exposure via any  job/substance/industry | 550 / 18,138 | 1 Ref |  | 1 Ref |  |
| No Exposure to smoke of all types | 427/14,838 | 0.96 | 0.84-1.09 | 0.96 | 0.84-1.09 |
| <10 years of Exposure | 6/250 | 0.82 | 0.37-1.84 | 0.82 | 0.37-1.84 |
| >10 years of Exposure | 5/232 | 0.68 | 0.28-1.65 | 0.68 | 0.28-1.65 |
| **Burning Coal Smoke** |  |  |  |  |  |
| No Exposure via any  job/substance/industry | 550 / 18,138 | 1 Ref |  | 1 Ref |  |
| No Exposure to burning coal smoke | 436/15,177 | 0.95 | 0.83-1.08 | 0.95 | 0.83-1.08 |
| <10 years of Exposure | 1/74 | 0.44 | 0.06-3.15 | 0.44 | 0.06-3.15 |
| >10 years of Exposure | 1/69 | 0.49 | 0.07-3.51 | 0.49 | 0.07-3.51 |
| **Burning Wood Smoke** |  |  |  |  |  |
| No Exposure via any  job/substance/industry | 550 / 18,138 | 1 Ref |  | 1 Ref |  |
| No Exposure to burning wood smoke | 436/15,209 | 0.95 | 0.84-1.09 | 0.95 | 0.83-1.08 |
| <10 years of Exposure | 0/44 | 0 | 0 | 0 | 0 |
| >10 years of Exposure | 2/67 | 1.03 | 0.25-4.13 | 0.99 | 0.25-4.00 |
| **Other Smoke** |  |  |  |  |  |
| No Exposure via any  job/substance/industry | 550 / 18,138 | 1 Ref |  | 1 Ref |  |
| No Exposure to other smoke | 405/13,968 | 0.96 | 0.84-1.10 | 0.95 | 0.83-1.09 |
| <10 years of Exposure | 19/698 | 0.96 | 0.61-1.53 | 0.94 | 0.59-1.50 |
| >10 years of Exposure | 14/654 | 0.76 | 0.45-1.29 | 0.74 | 0.43-1.26 |
| **Pesticides** |  |  |  |  |  |
| No Exposure via any  job/substance/industry | 550 / 18,138 | 1 Ref |  | 1 Ref |  |
| No Exposure to pesticides | 435/15,086 | 0.96 | 0.84-1.10 | 0.95 | 0.83-1.08 |
| <10 years of Exposure | 0/60 | 0 | 0 | 0 | 0 |
| >10 years of Exposure | 3/174 | 0.67 | 0.22-2.09 | 0.66 | 0.22-2.10 |
| **Coal/Tar/Soot** |  |  |  |  |  |
| No Exposure via any  job/substance/industry | 550 / 18,138 | 1 Ref |  | 1 Ref |  |
| No Exposure to coal/tar/soot | 438/15,293 | 0.95 | 0.83-1.09 | 0.94 | 0.83-1.08 |
| <10 years of Exposure | 0/18 | 0 | 0 | 0 | 0 |
| >10 years of Exposure | 0/9 | 0 | 0 | 0 | 0 |
| **Chemical Solvents** |  |  |  |  |  |
| No Exposure via any  job/substance/industry | 550 / 18,138 | 1 Ref |  | 1 Ref |  |
| No Exposure to chemical solvents | 410/14,372 | 0.95 | 0.83-1.08 | 0.94 | 0.82-1.08 |
| <10 years of Exposure | 11/476 | 0.79 | 0.43-1.44 | 0.78 | 0.43-1.43 |
| >10 years of Exposure | 17/472 | 1.14 | 0.70-1.86 | 1.13 | 0.69-1.84 |
| **Dyes/Dyestuffs** |  |  |  |  |  |
| No Exposure via any  job/substance/industry | 550 / 18,138 | 1 Ref |  | 1 Ref |  |
| No Exposure to dyes and dyestuffs | 433/15,163 | 0.95 | 0.83-1.08 | 0.94 | 0.87-1.08 |
| <10 years of Exposure | 1/76 | 0.4 | 0.06-2.83 | 0.39 | 0.06-2.80 |
| >10 years of Exposure | 81 | 1.47 | 0.55-3.93 | 1.48 | 0.55-3.96 |
| **Cutting oils** |  |  |  |  |  |
| No Exposure via any  job/substance/industry | 550 / 18,138 | 1 Ref |  | 1 Ref |  |
| No Exposure to cutting oils | 432/15,108 | 0.95 | 0.83-1.08 | 0.94 | 0.83-1.08 |
| <10 years of Exposure | 2/121 | 0.56 | 0.14-2.25 | 0.55 | 0.14-2.22 |
| >10 years of Exposure | 4/91 | 1.38 | 0.52-3.70 | 1.35 | 0.50-3.63 |
| **Paints** |  |  |  |  |  |
| No Exposure via any  job/substance/industry | 550 / 18,138 | 1 Ref |  | 1 Ref |  |
| No Exposure to paint | 435/15,151 | 0.95 | 0.84-1.09 | 0.95 | 0.83-1.08 |
| <10 years of Exposure | 2/100 | 0.7 | 0.17-2.81 | 0.69 | 0.17-2.79 |
| >10 years of Exposure | 1/69 | 0.5 | 0.07-3.60 | 0.49 | 0.07-3.50 |
| **JOB TITLE** |  |  |  |  |  |
| **Welder** |  |  |  |  |  |
| No Exposure via any  job/substance/industry | 550 / 18,138 | 1 Ref |  | 1 Ref |  |
| No Exposure as a welder | 438/15,303 | 0.95 | 0.83-1.09 | 0.95 | 0.83-1.09 |
| <10 years of Exposure | 0/10 | 0 | 0 | 0 | 0 |
| >10 years of Exposure | 0/7 | 0 | 0 | 0 | 0 |
| **Textile Machinist** |  |  |  |  |  |
| No Exposure via any  job/substance/industry | 550 / 18,138 | 1 Ref |  | 1 Ref |  |
| No Exposure working with textile machines | 437/15,293 | 0.95 | 0.83-1.08 | 0.94 | 0.83-1.08 |
| <10 years of Exposure | 1/13 | 2.48 | 0.35-17.7 | 2.5 | 0.35-17.8 |
| >10 years of Exposure | 0/14 | 0 | 0.00-0.00 | 0 | 0.00-0.00 |
| **Other Mechanic** |  |  |  |  |  |
| No Exposure via any  job/substance/industry | 550 / 18,138 | 1 Ref |  | 1 Ref |  |
| No Exposure as a mechanic | 436/15,251 | 0.95 | 0.83-1.08 | 0.94 | 0.83-1.08 |
| <10 years of Exposure | 1/32 | 0.99 | 0.14-7.05 | 0.99 | 0.14-7.04 |
| >10 years of Exposure | 1/37 | 0.91 | 0.13-6.50 | 0.9 | 0.1-6.42 |
| **Cotton Spinner** |  |  |  |  |  |
| No Exposure via any  job/substance/industry | 550 / 18,138 | 1 Ref |  | 1 Ref |  |
| No Exposure as a cotton spinner | 434/15,181 | 0.95 | 0.83-1.08 | 0.94 | 0.83-1.08 |
| <10 years of Exposure | 3/98 | 0.96 | 0.31-3.00 | 0.96 | 0.31-2.99 |
| >10 years of Exposure | 1/41 | 0.8 | 0.11-5.65 | 0.81 | 0.11-5.75 |
| **Painter** |  |  |  |  |  |
| No Exposure via any  job/substance/industry | 550 / 18,138 | 1 Ref |  | 1 Ref |  |
| No Exposure as a painter | 438/15,305 | 0.65 | 0.83-1.08 | 0.94 | 0.83-1.08 |
| <10 years of Exposure | 0/13 | 0 | 0 | 0 | 0 |
| >10 years of Exposure | 0/2 | 0 | 0 | 0 | 0 |
| **Textile Dyer** |  |  |  |  |  |
| No Exposure via any  job/substance/industry | 550 / 18,138 | 1 Ref |  | 1 Ref |  |
| No Exposure as a textile dyer | 438/15,312 | 0.95 | 0.83-1.08 | 0.94 | 0.83-1.08 |
| <10 years of Exposure | 0/6 | 0 | 0 | 0 | 0 |
| >10 years of Exposure | 0/2 | 0 | 0 | 0 | 0 |
| **Machinist** |  |  |  |  |  |
| No Exposure via any  job/substance/industry | 550 / 18,138 | 1 Ref |  | 1 Ref |  |
| No Exposure as a machinist | 438/15,310 | 0.95 | 0.83-1.08 | 0.94 | 0.83-1.08 |
| <10 years of Exposure | 0/3 | 0 | 0 | 0 | 0 |
| >10 years of Exposure | 0/7 | 0 | 0 | 0 | 0 |
| **Printer** |  |  |  |  |  |
| No Exposure via any  job/substance/industry | 550 / 18,138 | 1 Ref |  | 1 Ref |  |
| No Exposure as a printer | 424/14,981 | 0.94 | 0.83-1.08 | 0.94 | 0.82-1.07 |
| <10 years of Exposure | 7/208 | 1.04 | 0.49-2.19 | 1.05 | 0.50-2.21 |
| >10 years of Exposure | 7/131 | 1.51 | 0.72-3.19 | 1.52 | 0.72-3.20 |
| **Tailor** |  |  |  |  |  |
| No Exposure via any  job/substance/industry | 550 / 18,138 | 1 Ref |  | 1 Ref |  |
| No Exposure as a tailor | 310/11,167 | 0.95 | 0.82-1.10 | 0.94 | 0.81-1.09 |
| <10 years of Exposure | 60/1973 | 0.97 | 0.74-1.27 | 0.97 | 0.74-1.27 |
| >10 years of Exposure | 68/2180 | 0.93 | 0.72-1.21 | 0.94 | 0.73-1.22 |
| **Janitor** |  |  |  |  |  |
| No Exposure via any  job/substance/industry | 550 / 18,138 | 1 Ref |  | 1 Ref |  |
| No Exposure as a janitor | 337/11,552 | 0.95 | 0.83-1.10 | 1 | 0.82-1.10 |
| <10 years of Exposure | 61/2136 | 1 | 0.76-1.31 | 1 | 0.75-1.28 |
| >10 years of Exposure | 40/1632 | 0.86 | 0.62-1.18 | 0.8 | 0.61-1.17 |
| **Driver** |  |  |  |  |  |
| No Exposure via any  job/substance/industry | 550 / 18,138 | 1 Ref |  | 1 Ref |  |
| No Exposure as a taxi driver | 435/15,208 | 0.95 | 0.83-1.08 | 0.94 | 0.83-1.08 |
| <10 years of Exposure | 1/52 | 0.64 | 0.09-4.54 | 0.63 | 0.09-4.50 |
| >10 years of Exposure | 2/60 | 1.09 | 0.27-4.39 | 1.05 | 0.26-4.23 |
| **Food Hawker** |  |  |  |  |  |
| No Exposure via any  job/substance/industry | 550 / 18,138 | 1 Ref |  | 1 Ref |  |
| No Exposure as a food hawker | 342/11,584 | 0.97 | 0.84-1.12 | 0.96 | 0.84-1.11 |
| <10 years of Exposure | 57/2139 | 0.91 | 0.69-1.21 | 0.9 | 0.68-1.19 |
| >10 years of Exposure | 39/1597 | 0.86 | 0.62-1.19 | 0.84 | 0.61-1.17 |
| **INDUSTRY** |  |  |  |  |  |
| **Manufacture Leather** |  |  |  |  |  |
| No Exposure via any  job/substance/industry | 550 / 18,138 | 1 Ref |  | 1 Ref |  |
| No Exposure to leather manufacturing  industry | 420/14844 | 0.94 | 0.82-1.07 | 0.93 | 0.82-1.07 |
| <10 years of Exposure | 10/291 | 1.17 | 0.63-2.20 | 1.15 | 0.61-2.16 |
| >10 years of Exposure | 8/185 | 1.47 | 0.73-2.97 | 1.44 | 0.71-2.90 |
| **Manufacture Furniture** |  |  |  |  |  |
| No Exposure via any  job/substance/industry | 550 / 18,138 | 1 Ref |  | 1 Ref |  |
| No Exposure to furniture manufacturing  industry | 436/ 15223 | 0.95 | 0.83-1.09 | 0.94 | 0.83-1.08 |
| <10 years of Exposure | 0/65 | 0 | 0 | 0 | 0 |
| >10 years of Exposure | 2/32 | 2.31 | 0.58-9.28 | 2.37 | 0.59-9.53 |
| **Cotton Textile** |  |  |  |  |  |
| No Exposure via any  job/substance/industry | 550 / 18,138 | 1 Ref |  | 1 Ref |  |
| No Exposure to cotton textile industry | 419/14743 | 0.95 | 0.83-1.08 | 0.94 | 0.82-1.08 |
| <10 years of Exposure | 8/366 | 0.67 | 0.33-1.36 | 0.67 | 0.33-1.35 |
| >10 years of Exposure | 11/211 | 1.51 | 0.83-2.75 | 1.49 | 0.82-2.72 |
| **Manufacture Electrical Parts** |  |  |  |  |  |
| No Exposure via any  job/substance/industry | 550 / 18,138 | 1 Ref |  | 1 Ref |  |
| No Exposure to manufacturing of electrical parts industry | 382/13338 | 0.96 | 0.84-1.10 | 0.96 | 0.84-1.10 |
| <10 years of Exposure | 39/1471 | 0.82 | 0.59-1.14 | 0.81 | 0.58-1.13 |
| >10 years of Exposure | 17/511 | 1.00 | 0.61-1.64 | 0.98 | 0.60-1.60 |
| **Lumber** |  |  |  |  |  |
| No Exposure via any  job/substance/industry | 550 / 18,138 | 1 Ref |  | 1 Ref |  |
| No Exposure to lumber industry | 430/15,095 | 0.95 | 0.83-1.08 | 0.94 | 0.82-1.07 |
| <10 years of Exposure | 7/176 | 1.37 | 0.65-2.89 | 1.37 | 0.65-2.90 |
| >10 years of Exposure | 1/49 | 0.67 | 0.09-4.79 | 0.66 | 0.09-4.73 |
| **Carpentry** |  |  |  |  |  |
| No Exposure via any  job/substance/industry | 550 / 18,138 | 1 Ref |  | 1 Ref |  |
| No Exposure to carpentry industry | 436/15,251 | 0.95 | 0.83-1.08 | 0.94 | 0.83-1.08 |
| <10 years of Exposure | 2/47 | 1.51 | 0.38-6.06 | 1.50 | 0.37-6.03 |
| >10 years of Exposure | 0/22 | 0.00 | 0.00 | 0.00 | 0.00 |
| **Manufacture Rubber** |  |  |  |  |  |
| No Exposure via any  job/substance/industry | 550 / 18,138 | 1 Ref |  | 1 Ref |  |
| No Exposure to rubber manufacturing  industry | 405/14,205 | 0.94 | 0.83-1.08 | 0.94 | 0.82-1.07 |
| <10 years of Exposure | 19/638 | 1.08 | 0.68-1.71 | 1.05 | 0.66-1.66 |
| >10 years of Exposure | 14/477 | 0.98 | 0.57-1.67 | 0.95 | 0.56-1.63 |
| **Manufacture Dyes** |  |  |  |  |  |
| No Exposure via any  job/substance/industry | 550 / 18,138 | 1 Ref |  | 1 Ref |  |
| No Exposure to dye manufacturing  industry | 438/15,311 | 0.95 | 0.83-1.08 | 0.94 | 0.83-1.08 |
| <10 years of Exposure | 0/4 | 0 | 0 | 0 | 0 |
| >10 years of Exposure | 0/5 | 0 | 0 | 0 | 0 |
| **Manufacture Paint** |  |  |  |  |  |
| No Exposure via any  job/substance/industry | 550 / 18,138 | 1 Ref |  | 1 Ref |  |
| No Exposure to paint manufacturing  industry | 438/15,308 | 0.95 | 0.83-1.08 | 0.94 | 0.83-1.08 |
| <10 years of Exposure | 0/6 | 0 | 0 | 0 | 0 |
| >10 years of Exposure | 0/6 | 0 | 0 | 0 | 0 |
| **Manufacture Plastics** |  |  |  |  |  |
| No Exposure via any  job/substance/industry | 550 / 18,138 | 1 Ref |  | 1 Ref |  |
| No Exposure to plastics industry | 417/14,739 | 0.94 | 0.82-1.08 | 0.93 | 0.82-1.07 |
| <10 years of Exposure | 16/420 | 1.28 | 0.78-2.11 | 1.28 | 0.78-2.11 |
| >10 years of Exposure | 5/161 | 0.99 | 0.41-2.39 | 0.97 | 0.40-2.04 |
| **Petroleum** |  |  |  |  |  |
| No Exposure via any  job/substance/industry | 550 / 18,138 | 1 Ref |  | 1 Ref |  |
| No Exposure to petroleum industry | 438/15,302 | 0.95 | 0.83-1.09 | 0.94 | 0.83-1.08 |
| <10 years of Exposure | 0/13 | 0 | 0 | 0 | 0 |
| >10 years of Exposure | 0/5 | 0 | 0 | 0 | 0 |
| **Metal Production** |  |  |  |  |  |
| No Exposure via any  job/substance/industry | 550 / 18,138 | 1 Ref |  | 1 Ref |  |
| No Exposure to metal production industry | 418/14,778 | 0.94 | 0.83-1.08 | 0.93 | 0.82-1.07 |
| <10 years of Exposure | 13/364 | 1.25 | 0.72-2.17 | 1.23 | 0.71-2.15 |
| >10 years of Exposure | 7/178 | 1.27 | 0.60-2.68 | 1.27 | 0.60-2.68 |
| **Construction** |  |  |  |  |  |
| No Exposure via any  job/substance/industry | 550 / 18,138 | 1 Ref |  | 1 Ref |  |
| No Exposure to construction | 421/14,578 | 0.95 | 0.83-1.09 | 0.95 | 0.83-1.08 |
| <10 years of Exposure | 11/451 | 0.96 | 0.53-1.75 | 0.95 | 0.52-1.73 |
| >10 years of Exposure | 6/291 | 0.82 | 0.37-1.85 | 0.82 | 0.37-1.85 |

Supplementary References

1. Behrens T, Lynge E, Cree I, et al. Occupational exposure to endocrine-disrupting chemicals and the risk of uveal melanoma. *Scand J Work Environ Heal*. 2012;38(5):476-483. doi:10.5271/sjweh.3265.

2. of IWG on the E. Arsenic, metals, fibres, and dusts. *humans/World Heal …*. 2012. http://www.ncbi.nlm.nih.gov/pubmed/23189751. Accessed November 15, 2016.

3. Choe S-Y, Kim S-J, Kim H-G, et al. Evaluation of estrogenicity of major heavy metals. 2003;312(1-3):15-21. doi:10.1016/S0048-9697(03)00190-6.

4. Davey J, Nomikos A. Arsenic as an endocrine disruptor: arsenic disrupts retinoic acid receptor–and thyroid hormone receptor–mediated gene regulation and thyroid hormone–. *Environmental*. 2008. http://pubmedcentralcanada.ca/articlerender.cgi?artid=122138. Accessed November 15, 2016.

5. Fielden MR, Wu Z-F, Sinal CJ, et al. Estrogen receptor- and aryl hydrocarbon receptor-mediated activities of a coal-tar creosote. *Environ Toxicol Chem*. 2000;19(5):1262-1271. doi:10.1002/etc.5620190507.

6. National Library of Medicine(U.S.). Tox Town: Toxic chemicals and environmental health risks where you live and work and play. Endocrine Disruptors. https://toxtown.nlm.nih.gov/text_version/chemicals.php?id=65. Published 2016.

7. ATSDR. *Toxicological Profile for Wood Creosote, Coal Tar Creosote, Coal Tar, Coal Tar Pitch, and Coal Tar Pitch Volatiles*.; 2002. https://www.atsdr.cdc.gov/toxprofiles/tp85.pdf.

8. Iavicoli I, Fontana L, Bergamaschi A. The Effects of Metals as Endocrine Disruptors. *J Toxicol Environ Heal Part B*. 2009;12(3):206-223. doi:10.1080/10937400902902062.

9. Dyer CA. 5 Heavy Metals as Endocrine-Disrupting Chemicals. *Humana Press*. 2007;(Endocrine-disrupting Chemicals):111-133.

10. Thompson D, Kriebel D, Quinn MM, Wegman DH, Eisen EA. Occupational exposure to metalworking fluids and risk of breast cancer among female autoworkers. *Am J Ind Med*. 2005;47(2):153-160. doi:10.1002/ajim.20132.

11. Mnif W, Hassine AIH, Bouaziz A, Bartegi A, Thomas O, Roig B. Effect of endocrine disruptor pesticides: a review. *Int J Environ Res Public Health*. 2011;8(6):2265-2303. doi:10.3390/ijerph8062265.

12. Mueller JG, Chapman PJ, Pritchard PH. Creosote-contaminated sites. Their potential for bioremediation. *Environ Sci Technol*. 1989;23(10):1197-1201. doi:10.1021/es00068a003.

13. Clapp RW, Jacobs MM, Howe GK. Carcinogens, Environmental. In: *International Encyclopedia of Public Health*. ; 2008:493-507. doi:10.1016/B978-012373960-5.00276-8.

14. Ekenga CC, Parks CG, Sandler DP. Chemical exposures in the workplace and breast cancer risk: A prospective cohort study. *Int J cancer*. 2015;137(7):1765-1774. doi:10.1002/ijc.29545.

15. International Agency for Research on Cancer. *IARC MONOGRAPHS ON THE EVALUATION OF CARCINOGENIC RISKS TO HUMANS Volume 99 : Some Aromatic Amines, Organic Dyes, and Related Exposures*.; 2010. http://monographs.iarc.fr/ENG/Monographs/vol99/.

16. Bazin I, Ibn Hadj Hassine A, Haj Hamouda Y, et al. Estrogenic and anti-estrogenic activity of 23 commercial textile dyes. *Ecotoxicol Environ Saf*. 2012;85:131-136. doi:10.1016/j.ecoenv.2012.08.003.

17. Georgescu B, Georgescu C, Dărăban S, Bouaru A, Paşcalău S. Heavy Metals Acting as Endocrine Disrupters. *Sci Pap Anim Sci Biotechnol*. 2011;44(2).

18. Brouwers MM, van Tongeren M, Hirst AA, Bretveld RW, Roeleveld N. Occupational exposure to potential endocrine disruptors: further development of a job exposure matrix. *Occup Environ Med*. 2009;66(9):607-614. doi:10.1136/oem.2008.042184.

19. Stoye D, Freitag W, eds. *Paints, Coatings and Solvents*. Weinheim, Germany: Wiley-VCH Verlag GmbH; 1998. doi:10.1002/9783527611867.

20. Hultengren M, Axelsson I, Johnsson S, Rosen G. Industrial surface coating of wood and associated health hazards. Exposure to formaldehyde and solvents during surface coating of wood with acid curing paints and laquers. *Arb och Haelsa*. 1992.

21. International Agency for Research on Cancer. *IARC Monographs on the Evaluation of Carcinogenic Risks to Humans Volume 48: Some Flame Retardants and Textile Chemicals, and Exposures in the Textile Manufacturing Industry*.; 1990. https://monographs.iarc.fr/ENG/Monographs/vol48/.

22. International Labour Organization. *International Hazard Datasheets on Occupation Ship-Engineer (Machinist)*. http://www.ilo.org/wcmsp5/groups/public/---ed_protect/---protrav/---safework/documents/publication/wcms_193081.pdf. Accessed November 15, 2016.

23. International Agency for Reasearch on Cancer. *IARC Monographs on the Evaluation of Carcinogenic Risk in Humans. Volume 45: Occupational Exposures in Petroleum Refining; Crude Oil and Major Petroleum Fuels*.; 1989. http://monographs.iarc.fr/ENG/Monographs/vol45/.

24. International Agency for Research on Cancer. *IARC Monographs on the Evaluation of Carcinogenic Risk to Humans. Volume 100F 35: OCCUPATIONAL EXPOSURE AS A PAINTER*.; 2012.

25. HABEL L. ., STANFORD JL, VAUGHAN TL, et al. Occupation and breast cancer risk in middle-aged women. *J Occup Environ Med*. 37(3):349-356. http://cat.inist.fr/?aModele=afficheN&cpsidt=3553508. Accessed June 18, 2015.

26. Miller-Schulze JP, Paulsen M, Toriba A, et al. Exposures to particulate air pollution and nitro-polycyclic aromatic hydrocarbons among taxi drivers in Shenyang, China. *Environ Sci Technol*. 2010;44(1):216-221. doi:10.1021/es802392u.

27. Rai R, Glass DC, Heyworth JS, Saunders C, Fritschi L. Occupational exposures to engine exhausts and other PAHs and breast cancer risk: A population-based case-control study. *Am J Ind Med*. 2016;59(6):437-444. doi:10.1002/ajim.22592.

28. Rane S. Street vended food in developing world: hazard analyses. *Indian J Microbiol*. 2011;51(1):100-106. doi:10.1007/s12088-011-0154-x.

29. Dixit S, Yadav A, Dwivedi PD, Das M. Toxic hazards of leather industry and technologies to combat threat: a review. *J Clean Prod*. 2015;87:39-49. doi:10.1016/j.jclepro.2014.10.017.

30. Vinzents P, Laursen B. A national cross-sectional study of the working environment in the Danish wood and furniture industry--air pollution and noise. *Ann Occup Hyg*. 1993;37(1):25-34. http://www.ncbi.nlm.nih.gov/pubmed/8460875. Accessed November 15, 2016.

31. Koh D, Chan G, Yap E. World at work: the electronics industry. *Occup Environ Med*. 2004;61(2):180-183. doi:10.1136/oem.2002.001073.

32. International Agency for Research on Cancer. *IARC Monographs on the Evaluation of Carcinogenic Risk to Humans. Volume 100F 36: Occupational Exposures in the Rubber Manufacturing Industry*.; 2012. http://monographs.iarc.fr/ENG/Monographs/vol100F/mono100F-36.pdf.

33. De Coster S, van Larebeke N, De Coster S, van Larebeke N. Endocrine-Disrupting Chemicals: Associated Disorders and Mechanisms of Action. *J Environ Public Health*. 2012;2012:1-52. doi:10.1155/2012/713696.

34. International Agency for Research on Cancer. *IARC Monographs on the Evaluation of Carcinogenic Riskd to Humans. Volume 47: Some Organic Solvents, Resin Monomers and Related Compounds, Pigments and Occupational Exposures in Paint Manufacture and Painting*.; 1989.

35. Verma Y, Suresh V, Singh R. Endocrinal toxicity of industrial solvents – A mini review. *Indian J Exp Biol*. 2009;47:537-549.

36. Brophy JT, Keith MM, Watterson A, et al. Breast cancer risk in relation to occupations with exposure to carcinogens and endocrine disruptors: a Canadian case–control study. *Environ Heal*. 2012;11(1):87. doi:10.1186/1476-069X-11-87.

37. Construction Safety Council. *Health Hazards in Construction*. https://www.osha.gov/dte/grant_materials/fy09/sh-19495-09/health_hazards_workbook.pdf. Accessed November 15, 2016.
